# HEV Infection as an Aetiologic Factor for Acute Hepatitis: Experience from a Tertiary Hospital in Bangladesh

**DOI:** 10.3329/jhpn.v27i1.3314

**Published:** 2009-02

**Authors:** Mamun-Al-Mahtab, Salimur Rahman, Mobin Khan, Fazal Karim

**Affiliations:** ^1^ Department of Hepatology, Bangabandhu Sheikh Mujib Medical University, Shahbagh, Dhaka 1000, Bangladesh; ^2^ Dhaka Mahanagar General Hospital, Dhaka, Bangladesh

**Keywords:** Hepatitis, Hepatitis E infections, Hepatitis E virus, Retrospective studies, Bangladesh

## Abstract

Acute hepatitis is seen sporadically round the year in Bangladesh. The incidence of acute viral hepatitis E increases after floods as this allows sewerage contamination of piped and groundwater. The aim of this retrospective study was to assess the burden of hepatitis E virus (HEV infection) in Bangladesh. Patients attending the Hepatology Unit III of the Bangabandhu Sheikh Mujib Medical University, during June 2004–December 2006, were included in the study. All viral markers were tested by enzyme-linked immunosorbent assay. The study population was divided in four groups. Group 1 included 144 patients with acute viral hepatitis. The inclusion criteria were: nausea and/or vomiting, loss of appetite, serum bilirubin >200 μmol/L, raised serum transaminases, and prothrombin time ≥3 seconds prolonged beyond control value. In Group 2, there were 31 pregnant women with acute viral hepatitis. All the patients had prodrome, icterus, raised serum bilirubin and raised serum transaminase levels. Group 3 included 23 patients presenting with fulminant hepatic failure. In Group 4, 69 patients with cirrhosis of liver were included. They presented with features of decompensation for the first time. The inclusion criteria were: patients with established cirrhosis with jaundice and/or ascites and/or hepatic encephalopathy. In Group 1, 58.33% of the 144 patients had acute viral hepatitis E. In Group 2, 45.16% of the pregnant women also had acute viral hepatitis E. HEV was responsible for 56.52% cases of fulminant hepatic failure in Group 3. In 21.7% cases in Group 4, decompensation of cirrhosis was due to HEV. Acute viral hepatitis E in the third trimester of pregnancy and HEV-induced fulminant hepatic failure were associated with 80% of mortality despite the best possible care. In this clinical context, acute viral hepatitis E is the leading cause of wide spectrum of liver disease ranging from severe acute viral hepatitis, fulminant hepatic failure, to decompensation of liver in cirrhotics in Bangladesh. Sewerage contamination of piped water following floods may contribute to the higher incidence of HEV infection.

## INTRODUCTION

Acute viral hepatitis E, a self-limiting disease presenting as acute, icteric hepatitis, is caused by hepatitis E virus (HEV). It is a small, non-enveloped RNA virus, icosahedral in shape, and 27-34 nm in diametre (1). HEV is commonly transmitted through the faecooral route by contaminated water. Bloodborne transmission and zoonotic reservoir of HEV have also been reported (2).

HEV has been responsible for major outbreaks of acute infection in developing countries of Asia, Africa, and Latin America over the last 50 years. The first documented epidemic of HEV was reported in New Delhi, India, in 1955-1956, and 29,300 people were affected (3). Outbreaks have since been reported from many countries of South and South-East Asia, Eastern Europe, and North and East Africa. The longest lasting outbreak was reported from South Xinjiang in Uighar region of China; the outbreak lasted for 20 months in 1986-1988. Over 119,000 cases were reported (4) while the Kanpur outbreak of India in 1991 recorded over 79,000 cases (5). Outbreaks of HEV usually coincide with heavy rains, resulting in flooding. This leads to sewage contamination of piped water through nearby sewerage lines or from contaminated surface-water. A similar outbreak was experienced in Dhaka city following the last major flooding in 2004. Almost 80% of Bangladesh was under flood-water at that time for over a month. The outbreak affected over 1,500 students of residential halls of the University of Dhaka (6). In the West and other developed countries, sporadic infections are seen in patients with a history of residence in or recent travel to endemic areas.

In the general population, HEV carries a low mortality of 0.5-4% (7). However, this figure approaches >75% in developing countries, such as Bangladesh, in the second/third trimester of pregnancy, and in patients with fulminant hepatic failure (7).

Detection of anti-HEV IgM still remains the main diagnostic marker of acute HEV infection (8). Anti-HEV IgM appears in the serum of infected patients at the onset of symptoms, precedes appearance of anti-HEV IgG, and wanes within 2-5 months after infection (9). HEV's RNA remains a more specific tool for the diagnosis of the infection; it, however, remains positive in serum for a short duration, and the test is also not commercially available.

## MATERIALS AND METHODS

HEV infection is common in Bangladesh. A previous study in Bangladesh has shown that the prevalence of anti-HEV IgM in our apparently-healthy population was 7.3% (10). In Pakistan, Hamid and colleagues from the Aga Khan Medical College in Karachi have reported a 17.5% prevalence of IgG to HEV in their population (11).

This is a retrospective study. Patients attending the Hepatology Unit III of the Bangabandhu Sheikh Mujib Medical University (BSMMU) during June 2004–December 2006 were included. All viral markers were tested by enzyme-linked immunosorbent assay (Abbott Labs, Chicago, USA). The viral markers that were tested included HBsAg, anti-HBc IgM, and HBeAg for hepatitis B virus (HBV), anti-HEV IgM for HEV, anti-HAV IgM for hepatitis A virus (HAV), anti-HCV for hepatitis C virus (HCV), and anti-CMV for cytomegalovirus (CMV). In cirrhotics, HBV's DNA was tested by polymerase chain reaction (PCR) (Taq Man PCR, Roche Molecular Systems, California, USA) having a lower limit of detection of 250 copies/mL. Serum bilirubin, transaminases, and albumin were tested using an autoanalyzer and prothrombin time by Quick's method. Tests were done at the Departments of Biochemistry, Hematology, and Virology of BSMMU maintaining high quality and assuring maximum possible accuracy of reports.

The study had three main foci: (a) whether HEV attributes a major aetiologic fraction of clinical acute viral hepatitis, (b) if there is any association between hepatic/cirrhotic decompression and HEV infection in this region, and (c) the prognosis in patients with HEV-attributed FHF/ALF seen in these contexts.

The study population (n=267) was divided into four groups. Group 1 included 144 patients with acute viral hepatitis. The inclusion criteria were: prodromal features, such as nausea and/or vomiting, loss of appetite, and serum bilirubin >200 μmol/L, raised serum transaminases, and prothrombin time ≥3 seconds prolonged beyond control value.

Group 2 included 31 pregnant women with acute viral hepatitis. All the patients had prodrome, icterus, raised serum bilirubin and raised serum transaminase levels.

Group 3 included 23 patients presenting with fulminant hepatic failure. Patients were diagnosed based on the history from attendants, clinical features, physical examinations, laboratory investigations, and imaging. Only those patients who presented with severe impairment of hepato-cellular function, i.e. encephalopathy, coagulopathy, and jaundice, within six months of onset of symptoms were included in the study. They were all previously healthy.

Finally, Group 4 had 69 patients with cirrhosis of liver. They presented with features of decompensation for the first time. The inclusion criteria were patients with established cirrhosis with jaundice and/or ascites with/without encephalopathy.

## RESULTS

Of the 144 patients in Group 1, 58.33% (n=84) had acute viral E hepatitis, 31.25% (n=45) had acute viral B hepatitis, 4.86% (n=7) had acute viral A hepatitis, 2.78% (n=4) had mixed infection with both hepatitis E and A viruses, and 0.69% (n=1) had acute CMV hepatitis. Hepatitis was cryptogenic in the remaining 2.07% (n=3) of the 144 patients.

In Group 2, of the 31 patients, 45.16% (n=14) had acute viral E hepatitis, 6.45% (n=2) had acute viral B hepatitis, and 9.38% (n=3) had mixed infection with both hepatitis E and B viruses. No virus marker could be detected in 38.71% (n=12) of the 31 patients. Overall, 54.84% (n=17) of the 31 patients had HEV infection while 16.12% (n=5) had infection with HBV.

In Group 3, of the 23 patients, 56.52% (n=13) had HEV infection, 34.78% (n=8) had infection due to HBV, and 8.7% (n=2) had a history of intake of hepatotoxic drugs or alcohol abuse.

Finally, in Group 4, of the 69 patients, 21.7% (n=15) were anti-HEV IgM-positive, 4.3% (n=3) had a history of recent haemorrhage from the upper gastrointestinal tract, and 4.3% (n=3) had a history of intake of hepatotoxic drugs or alcohol abuse. No specific cause for decompensation of liver could be identified in the remaining 69.6% (n=48) of the patients. Decompensation in these cases probably resulted from the progression of cirrhosis.

Except for those included in Group 4, the patients had no pre-existing liver disease as revealed from their records. Patients belonging to Group 1 and Group 2 were conservatively managed and carefully followed up for the detection and management of complications early.

In Group 2, the patients received conservative and supportive management with barrier nursing as far as practicable. Temperature and blood pressure were monitored at regular intervals, and an input-output chart was maintained to avoid volume overload. Injection ceftriaxone was administered intravenously at a dose of 1 g twice daily to guard against infections, and continuous intravenous glucose infusion was given to prevent fatal hypoglycaemia. Intravenous cannula was replaced on every third day. Patients with features of bleeding received fresh frozen plasma transfusion. Fresh frozen plasma transfusion was also given before and after labour or caesarian section.

In Group 3, patients developed severe impairment of hepato-cellular function within less than six months of onset of their symptoms. This impairment of liver function was evidenced by encephalopathy, coagulopathy, and jaundice. The patients received conservative and supportive management with barrier nursing as far as practicable. Temperature and blood pressure were monitored at regular intervals, and an input-output chart was maintained to avoid volume overload. Nasogastric tube feeding and continuous catherization was constituted. Injection ceftriaxone was administered intravenously at a dose of 1 g twice daily to guard against infections, and continuous intravenous glucose infusion was given to prevent fatal hypoglycaemia. Intravenous cannula was replaced on every third day. Intravenous dopamine infusion was given to prevent hypotension and renal failure. In patients with features of hepato-renal syndrome, intravenous albumin infusion and dopamine and nor-adrenaline drips were given. Patients with features of bleeding received fresh frozen plasma transfusion. Fresh frozen plasma transfusion was also given before any invasive intervention. Intravenous mannitol was infused rapidly to prevent cerebral oedema in suspected patients.

In Group 4, patients were conservatively managed with antibiotics, non-selective beta-blocker, diu-retics, albumin infusion, proton pump inhibitor, lactulose, vitamin K injection, and specific treatment directed towards aetiology of cirrhosis. For instance, those with HBV-related cirrhosis received oral antivirals if they tested positive for HBV's DNA by PCR.

Despite such measures, the outcome was generally fatal with high mortality recorded in patients in Group 2 and Group 3. HEV-related patients with fulminant hepatic failure had a mortality of 80%. Mortality was also 80% in the third trimester of pregnancy; however, none of the patients died, who were in their first or second trimester. The only factor that might have influenced HEV-related mortality in pregnancy appears to be the trimester of pregnancy, and other factors, such as age, socioeconomic background, or parity, had no effect.

## DISCUSSION

We observed that HEV was an important attributor to the aetiologic fraction of clinical acute viral hepatitis (Table). This observation is not different from other studies done in our region, namely in India, where HEV infection accounts for 50-70% of all cases of sporadic viral hepatitis (11,12). A group of researchers in India concluded from their study of 76 pregnancies with acute hepatitis that HEV was the commonest cause (49.6%) (12). HBV, HAV, HCV, HDV, and non-A, non-E were responsible for 15%, 1.5%, 1.7%, 1.5%, and 30.7% of cases respectively. They also found that, in the third trimester, HEV infection was associated with 22.2% of mortality compared to only 2.8% in non-pregnant women (13). Another Indian study also made similar observation. In their series of 62 pregnant patients, they found that 45.2% had HEV infection (14). The same researchers also found that HEV was responsible for 81% of cases with fulminant hepatic failure and 26.9% death cases in their patients (14). In Turkey, a study has shown that 12.6% (n=245) of acute hepatitis cases were HEV-related (15).

**Table.** Burden of HEV-induced liver disease in Bangladesh

Acute hepatitis	Results
HEV hepatitis in pregnant women (%)	58.33 (n=31)
HEV hepatitis in non-pregnant women (%)	45.16 (n=144)
Age (years)	8-82 (mean 32)
Male : female (non-pregnant) ratio	96:48
Mortality (pregnant: non-pregnant) (%)	80:0
Fulminant hepatic failure	
HEV-related	56.52 (n=23)
Age (years)	27-63 (mean 39)
Male : female ratio	17:6
Mortality (%)	80
Decompensation of cirrhosis	
HEV-related	21.7 (n=69)
Age (years)	23-61 (mean 37)
Male : female ratio	48:21
Mortality (%)	5

HEV=Hepatitis E virus

We also identified HEV as a principal cause of fulminant hepatic failure/acute liver failur (ALF) in our study population (Table). Similar observation has been made in different studies done in our region. An earlier study in Bangladesh analyzed sera of 22 patients with fulminant hepatic failure (10). Anti-HEV IgM was detected in 63.6% and HBsAg in 35.7% of cases. Analysis of sera from 273 apparently-healthy individuals revealed that 7.3% of the samples were positive for anti-HEV IgG. HEV infection was observed in 83.3% of HBV carriers (10). Researchers from India have studied 180 cases of fulminant hepatic failure to conclude that HEV is responsible for 43.9% of cases and HAV for 2.1%, HBV for 13.9%, HCV for 7.2%, and non-A, non-E agents for 31.1% of patients (16). Another study in India also revealed similar data showing that, of 102 cases of fulminant hepatic failure, HEV remained the commonest aetiologic agent, followed by HBV, HAV, and HCV (17). A handful of cases were due to drugs and Wilson's disease while no cause could be identified in 15 cases (17).

Finally, we found the association between hepatic/cirrhotic decompression and HEV infection (Table). In one of our works where we studied 139 patients with cirrhosis of liver, we found that HBV was the commonest cause of cirrhosis being responsible for 61.15% of the cases, followed by HCV (30.94%) while 3.6% of the cases were cryptogenic, possibly reflecting occult HBV infection and non-alcoholic steato-hepatitis (NASH) (18). Results of other studies also showed that HEV was the leading cause of decompensation of liver in patients with cirrhosis in the developing world. In a study in India, 72 patients with recently-decompensated HBV-related cirrhosis of liver were included. Tests for hepatitis A to E, HBV DNA, and HIV-1 and HIV-2 were performed (20). Extrinsic causes of decompensation were found in 35.6% of patients. It was observed that anti-HEV IgM was positive in 8.64%, anti-HDV IgM in 10.17%, and anti-HAV IgM in 3.34% of patients while 3.34% were positive for both anti-HDV IgM and anti HEV IgM. Intrinsic causes for decompensation, e.g. HBV reactivation and HCC, were found in 11.9% of patients, and 52.61% had neither any intrinsic nor extrinsic cause. It was, thus, concluded that HEV, followed by HDV, are the most common causes of decompensation of liver in cirrhotics in India (20). Another study in India included 10 patients with recently-decompensated cirrhosis of liver (21). Of them, five had alcohol, two had HBV, and one had HCV as the aetiology for their cirrhosis. The remaining two were suffering from cryptogenic cirrhosis of liver. The patients were tested for HAV, HBV, HCV, and HEV. All the 10 patients tested positive for anti-HEV IgM and negative for the remaining viruses (21). Researchers from North India shared similar experience. In their work, Arvind *et al.* studied 32 patients with decompensated cirrhosis of liver, of whom 44% tested positive for anti-HEV IgM (22). On the contrary, anti-HEV IgM was positive in only 6% of patients with compensated cirrhotics. They concluded that HEV is a frequent cause of decompensation of cirrhotic liver (22).

Another study from Karachi, Pakistan, documented four patients with recently-decompensated cirrhosis of liver, all of whom tested positive for anti-HEV IgM (11). The study found that anti-HEV IgG was positive in 17.5% of 233 patients with compensated cirrhosis, which is similar to 17.7% of healthy blood donors who also tested positive for anti-HEV IgG (11). A Nepalese study that recruited 12 patients with decompensated cirrhosis of liver found that all of them tested positive for anti-HEV IgM (23). Of them, eight were alcoholic, one HBV-related, one HCV-related, one cardiac, one Budd Chiari-Syndrome, and one cryptogenic cirrhosis of liver (23). Finally, a very recent work in India, where 107 decompensated cirrhotics and 200 controls were studied, found that HEV's RNA was positive in 28% of cirrhotic patients compared to only 4.5% of controls (24). Seventy percent with and 27% without rapid decompensation had HEV infection. Mortality between HEV-infected and non-infected decompensated cirrhotic patients at four weeks (43% vs 22%) and at 12 months (70% vs 30%) was different (24).

Our study has certain limitations. It is a retrospective hospital-based study. Besides, although we had patients from all over the country, most of them came from around Dhaka. The sample size was also not large. The data are, therefore, referral-biased and allow only an insight into the clinical burden of HEV infections among the urban population of the capital city of Bangladesh. However, our findings are similar to those reported by colleagues from our region.

Acute viral E hepatitis is an important cause of wide spectrum of clinically-presenting liver disease ranging from severe acute viral hepatitis, fulminant hepatic failure, to decompensation of liver in cirrhotics in Bangladesh. Poor sanitation and hygienic conditions, lack of safe drinking-water supply, and overcrowding may all be responsible for the high prevalence of HEV infection. Besides, sewerage contamination of piped water following floods may also contribute to the higher incidence of HEV infection. Currently, there is no specific treatment for HEV infection. Treatment is supportive. Similarly, no vaccine against HEV is commercially available to date. Therefore, improvement of personal hygiene, ensuring supply of safe drinking-water, and, most importantly, raised public awareness remain important to guard against this virus for the time-being. Keeping in mind the significant morbidity and also mortality associated with HEV infection in certain settings, this issue needs to be addressed with due importance, and physicians, NGOs, media, and political and community leaders should all discharge their respective responsibilities in this regard.

## References

[1] Margolis HS, Alter MJ, Halder SC, Evans AS, Kaslow RA (1997). Viral hepatitis. Viral infections of humans: epidemiology and control.

[2] Favorov M, Nazarova O, Margolis HS Is hepatitis E an emerging zoonotic disease? Presented at the Second International Conference on Emerging Zoonoses, Strasbourg, France, November 5-9, 1998. http://epirev.oxfordjournals.org/cgi/reprint/21/2/162.pdf.

[3] Vishwanathan R (1957). Infectious hepatitis in Delhi (1955-56): a critical study. Epidemiology. Indian J Med Res.

[4] Zuang H, Cao XY, Liu CB, Shikata T, Purcell RH, Uchida T (1991). Enterically transmitted non-A, non-B hepatitis in China. Viral hepatitis C, D, and E: proceedings of the International Meeting on Non-A, Non-B Hepatitis.

[5] Ray R, Aggarwal R, Salunke PN, Mehrotra NN, Talwar GP, Naik SR (1991). Hepatitis E virus genome in stools of hepatitis patients during large epidemic in north India. Lancet.

[6] Mahtab MA, Khan M, Alam K, Rahman S, Ahmad N, Mamun AA (2006). Hepatitis E virus is a leading cause of decompensation of liver in cirrhotic patients in Bangladesh. Liver Int.

[7] Krawczynski K (1993). Hepatitis E. Hepatology.

[8] Clayson ET, Myint KS, Snitbhan R, Vaughn DW, Innis BL, Chan L (1995). Viremia, fecal shedding, and IgM and IgG responses in patients with hepatitis E. J Infect Dis.

[9] Bradley DW (1995). Hepatitis E virus: a brief review of the biology, molecular virology, and immunology of a novel virus. J Hepatol.

[10] Sheikh A, Sugitani M, Kinukawa N, Moriyama M, Arakawa Y, Komiyama K (2002). Hepatitis E virus infection in fulminant hepatitis patients and an apparently healthy population in Bangldesh. Am J Trop Med Hyg.

[11] Hamid SS, Atiq M, Shehzad F, Yasmeen A, Nissa T, Salam A (2002). Hepatitis E virus superinfection in patients with chronic liver disease. Hepatology.

[12] Khuroo MS, Rustgi VK, Dawson GJ, Mushahwar IK, Yattoo GN, Kamili S (1994). Spectrum of hepatitis E virus infection in India. J Med Virol.

[13] Khuroo MS, Duermeyer W, Zargar SA, Ahanger MA, Shah MA (1983). Acute sporadic non-A, non-B hepatitis in India. Am J Epidemiol.

[14] Khuroo MS, Kamili S (2003). Aetiology, clinical course and outcome of sporadic acute viral hepatitis in pregnancy. J Viral Hep.

[15] Kumar A, Beniwal M, Kar P, Sharma JB, Murthy NS (2004). Hepatitis E in pregnancy. Int J Gynaecol Obstet.

[16] Khuroo MS, Kamili S (2003). Aetiology and prognostic factors in acute liver failure in India. J Viral Hep.

[17] Bansal N, Sud R, Arora A, Kumar M, Saigal S, Puri R (2005). Acute liver failure: aetiology, spectrum and predictors of survival on multivariate analysis. Indian J Gastroenterol.

[18] Afroz S, Mahtab MA, Rahman S, Khan M (2007). Hepatitis B virus is the leading cause of cirrhosis of liver in Bangladesh. Hepatol Int.

[19] Cevrioglu AS, Altindis M, Tanir HM, Aksoy F (2004). Investigation of the incidence of hepatitis E virus among pregnant women in Turkey. J Obstet Gynaecol Res.

[20] Das K, Sharma BC, Sarin SK (2005). Causes and profile of acute decompensation in hepatitis B related chronic liver disease in India. Indian J Gastroenterol.

[21] Monga R, Garg S, Tyagi P, Kumar N (2004). Superimposed acute hepatitis E infection in patients with chronic liver disease. Indian J Gastroenterol.

[22] Kumar A, Aggarwal R, Naik SR, Saraswat V, Ghoshal UC, Naik S (2004). Hepatitis E virus is responsible for decompensation of chronic liver disease in an endemic region. Indian J Gastroenterol.

[23] Sudhamshu KC (2006). Effects of hepatitis E virus infection in patients with chronic liver disease. J Gastroenterol Hepatol.

[24] Kumar Acharya S, Kumar Sharma P, Singh R, Kumar Mohanty S, Madan K, Kumar Jha J (2006). Hepatitis E virus (HEV) infection in patients with cirrhosis is associated with rapid decompensation and death. J Hepatol.

